# Extinction and beyond: an expanded framework for exposure and response prevention for obsessive-compulsive disorder

**DOI:** 10.3389/fpsyg.2024.1331155

**Published:** 2024-05-31

**Authors:** Hannah Berg, Ryan D. Webler, Samuel Klein, Matt G. Kushner

**Affiliations:** ^1^Department of Psychology, University of Minnesota Twin Cities, Minneapolis, MN, United States; ^2^Laureate Institute for Brain Research, Tulsa, OK, United States; ^3^Department of Psychiatry, University of Minnesota Twin Cities, Minneapolis, MN, United States

**Keywords:** exposure therapy, anxiety disorders, obsessive-compulsive disorder, extinction, behavioral therapy

## Abstract

Exposure therapy is a first-line, empirically validated treatment for anxiety, obsessive-compulsive, and trauma-related disorders. Extinction learning is the predominant theoretical framework for exposure therapy, whereby repeated disconfirmation of a feared outcome yields fear reduction over time. Although this framework has strong empirical support and substantial translational utility, extinction learning is unlikely to be the sole process underlying the therapeutic effects of exposure therapy. In our clinic, we commonly treat obsessive-compulsive disorder (OCD) patients successfully with exposure therapy even when some or all of their feared outcomes are not amenable to disconfirmation and, by extension, to extinction learning. Herein, we present a generic clinical vignette illustrating a commonly encountered feared outcome in OCD that cannot be disconfirmed through exposure (damnation resulting from blasphemous thoughts). We describe two specific non-extinction-based strategies we commonly employ in such cases, and we associate these strategies with known change mechanisms that might account for their effectiveness: (1) non-associative habituation to aversive stimuli, and (2) fear-memory elicitation and subsequent reconsolidation. We discuss the limitations inherent in the reverse-translational approach taken and its opportunities for expanding the framework of exposure therapy.

## 1 Introduction

**Figure 1:**
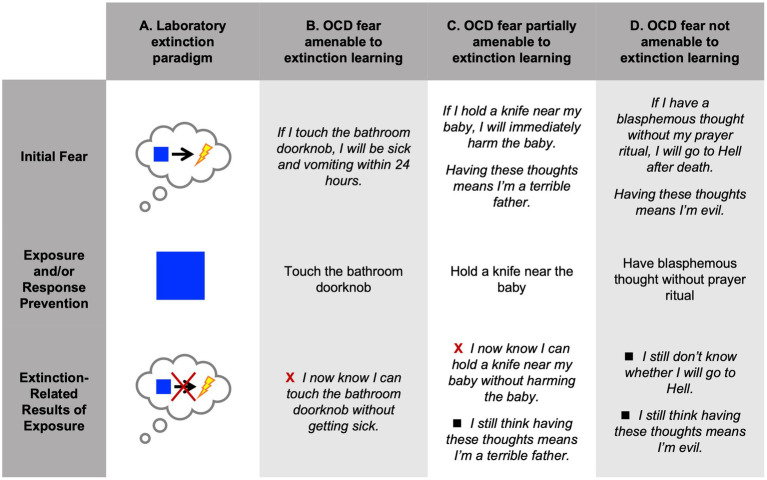
**(A)** In a traditional laboratory extinction paradigm, the initial appraisal that a neutral stimulus (blue square) will be followed by an aversive outcome (shock) is usually acquired through conditioning. Repeated exposure to the blue square without shock is expected to result in new experiential learning in the form of extinction, evidenced by declining fear responses. **(B)** In a common presentation of OCD, repeatedly touching the bathroom doorknob would lead to extinction if the patient expects an aversive outcome and unambiguously does not experience it. **(C)** In another common presentation of OCD, fears of causing harm are readily disconfirmed through repeated exposures to the perceived “dangerous” situation. Of note, the patient may still harbor additional fears about the meaning of his thoughts, which would not be directly disconfirmed through the exposure described. **(D)** The feared outcome of going to Hell after death cannot be disconfirmed through exposure; a patient would be unlikely to experience extinction of this core fear. The patient’s fears about the meaning of her thoughts are also unlikely to be disconfirmed through simply omitting the compulsive ritual. Red X’s indicate successful extinction learning. Black squares represent absence of extinction learning.

Exposure therapy is a first-line behavioral treatment for anxiety, obsessive-compulsive, and trauma-related disorders that involves repeated contact with feared stimuli, contexts, and scenarios (Foa and McLean, 2016; Reid et al., 2021; McLean et al., 2022). Exposure procedures were largely inspired by a cross-species classical conditioning literature showing that acquired threat responses can be ‘extinguished’ when threat cues (conditioned stimuli, CS+) are repeatedly experienced in the absence of an anticipated aversive outcome (unconditioned stimulus, US; Figure 1, column A) (Graham and Milad, 2011).

Although extinction (Table 1) is the prevailing theoretical framework for exposure-based therapy practices (Foa and McLean, 2016; Craske et al., 2022), there is increasing recognition that additional psychological change processes contribute to the therapeutic effects of exposure. For decades, experimental researchers have voiced concerns about the translational limitations of experimental paradigms that model extinction learning (Rachman, 1977; Poulton and Menzies, 2002; Mineka and Zinbarg, 2006; De Houwer, 2020) and avoidance (Krypotos et al., 2015). Echoing these concerns in the domain of clinical fear reduction, recent reviews have advised experimental researchers to “mind the gap” between fear reduction through extinction in the laboratory and the treatment of fear through exposure in the clinic (Carpenter et al., 2019) and urged the development of refined therapeutic models (Chowdhury and Khandoker, 2022; Nihei et al., 2023). Notably, Craske et al. (2008) suggested that, in addition to extinction learning, investigators should consider “other clinically relevant cognitive-emotional processes” that contribute to the beneficial effects of exposure therapy. A central conclusion of these reviews is well-captured by Kredlow et al. (2022) who noted that “…many factors that are central to exposure therapy in the clinic are not sufficiently modeled in the laboratory.”

**Table 1:** Definitions of terms.

Extinction	A decline in fear, or a change in threat-related behavior, caused by the repeated disconfirmation of an expected negative outcome. Includes classical extinction, in which a feared stimulus is confronted in the absence of an aversive stimulus expected to accompany it, and operant extinction, in which a feared behavior (or omission of a safety behavior) is performed in the absence of an expected aversive outcome. This can take place regardless of whether the original fear association was acquired through conditioning or otherwise.
Non-associative habituation	A decline in fear caused by the repeated presentation of an aversive stimulus.

These concerns align with our experience as practicing behavioral therapists who treat obsessive-compulsive disorder (OCD) with exposure and response prevention (ExRP). Our experience supports the conclusion that the extinction learning framework is insufficient to fully account for the therapeutic change brought about by successful exposure therapy. Taken together with the assertion (Abramowitz, 2013) that the practice of exposure therapy benefits from a solid understanding of the underlying theory, this conclusion points to a need to re-examine the extinction learning model alongside clinical insights, empirical evidence, and alternative models. This is particularly evident in a subset of our patients who present with fears that are not formally amenable to extinction learning, because the occurrence of their feared outcome(s) cannot be disconfirmed through exposure.

Under the extinction learning model, fear reduction occurs when a negative expectancy is repeatedly disconfirmed through experience. This model neatly accounts for the success of exposure therapy in many instances. Consider the classic OCD presentation of patients who fear they will become sick and vomit shortly after touching a presumably contaminated surface such as a public bathroom door handle, and who avoid touching such surfaces whenever possible and wash excessively following any contact with them. In response to these fears, the patient avoids touching such surfaces whenever possible and washes excessively following any contact with them. As illustrated in Figure 1 (column B), treatment for such a patient would entail touching bathroom door handles (and other public surfaces) without washing. Repeating this exercise in the absence of both illness and compulsive washing induces extinction learning, such that the patient no longer expects that illness will result from contact with objects in public spaces.

We also frequently treat patients whose symptoms do *not* lend themselves to an extinction learning framework, or do so only partially. Consider another common OCD presentation: patients who experience intrusive thoughts of harming loved ones and who, as a result, avoid approaching loved ones and experience intense guilt and shame for having these aggressive thoughts: e.g., “I must be a terrible person.” Repeated exposure exercises in which the patient stands near their loved one without causing harm, perhaps while holding a kitchen knife or other potentially dangerous items, should disconfirm the feared outcome and, hence, lead to extinction of the association between having harm-related thoughts and engaging in harm-related behaviors. However, the patient’s disturbing interpretation of their intrusive thoughts (e.g., “I must be a terrible person”) is not amenable to disconfirmation through exposure (Figure 1, column C).

While patients who fear that they will cause harm would be expected to obtain partial relief through extinction learning, other patients that we frequently encounter would not. Consider, for example, patients with religious scrupulosity symptoms who fear that their intrusive blasphemous thoughts, in the absence of a prayer ritual, will result in damnation after death (Figure 1, column D). Because the primary feared outcome is expected to occur after death, it is not amenable to disconfirmation and, hence, cannot be extinguished through exposure. Such patients might also harbor fear and uncertainty (Morriss et al., 2021) about the meaning of these thoughts; these fears would also not be amenable to disconfirmation through exposure.

As practicing behavioral therapists who primarily treat OCD through ExRP, we have developed several strategies for treating patients’ feared outcomes that cannot be disconfirmed through exposure. Refining these approaches over years of practice to address a clinical need has stimulated our thinking about the therapeutic processes involved in exposure therapy more broadly. This has evolved into the present reverse translational endeavor in which we attempt to formalize our non-extinction-based techniques, and relate them to the existing theoretical and empirical literature. More specifically, we integrate the extinction framework with propositional expectancy theory (Lovibond, 2006), which describes experiential and non-experiential sources of learning, and emotional processing theory (Rachman, 1980; Foa and Kozak, 1986), which posits a “fear structure” that can be altered through strategic methods of incorporating corrective information.

To contextualize the issues and techniques introduced, we describe a clinical vignette (Box 1) representing a composite of many cases treated in our clinic, as a generic case description. The symptoms described – ego dystonic blasphemous thoughts associated with thoughts and images of eternal damnation – are common in OCD and used here to illustrate our exposure treatment approach in such cases. We chose this vignette to highlight a situation where the importance of considering non-extinction-based approaches is particularly clear; i.e., when the primary fear allows for little or no disconfirmation through exposure. Following this, we describe two putative mechanisms, grounded in existing empirical and theoretical literature, that map onto the therapeutic action of the non-extinction-based interventions we describe.

BOX 1: **Generic Clinical Vignette of Treatment for Obsessional Fears that Cannot be Disconfirmed Through Exposure**

**Symptom Overview**

The patient reports daily ego dystonic sacrilegious thoughts and images. The patient reports reciting an idiosyncratic prayer to neutralize the negative consequences of having committed blasphemy. The prayer is only partially effective in alleviating the fears since the patient believes that having these thoughts and images may indicate a sinful nature deserving damnation even with the ritualized prayer.

**Imaginal Exposure to Blasphemous Thoughts**

Based on a detailed clinical interview, the therapist develops a hierarchy of exposure exercises with ritual prevention and provides the patient with a description of the treatment.

The therapist obtains the patient’s agreement to make an audio recording of the imaginal exposure narrative so that they can repeat the exercise outside of the session (“homework”).

In the exposure, the therapist describes specific blasphemous thoughts and describes specific scenes of being damned as a result, both of which are based on details obtained from the patient in the pre-treatment interview. The patient is asked to repeat some of the specific blasphemous statements in the narration and to occasionally rate their degree of discomfort.

**Introduction of New Information**

After about five minutes of narration during which the patient’s fears are strongly activated (as indexed by subjective ratings), the therapist introduces new information meant to challenge and modify the patient’s interpretation (meaning) of the upsetting thoughts. After confirming that the patient believes that having blasphemous thoughts means they are sinful and deserving of damnation, the therapist offers an alternative interpretation along the following lines.

*“You’ve said you fear that your upsetting thoughts mean you are inherently sinful and deserving of damnation, but it seems you’ve never considered that they could be a sign of just how much you despise damnation and love God. This view is easier to understand if you consider how the brain’s primitive safety system operates. This brain system – we can call it your ‘safety brain’ – tries to keep you from doing dangerous things by showing you the terrible consequences these things can cause. It’s like your brain shows you a horrible movie to motivate you to avoid these bad outcomes. This sort of process is not uncommon. For example, many people report that when they are walking near the edge of a cliff or a bridge, they have a vivid image of their jumping off, flying through the air in terror, and hitting the ground where they experience horrible pain just before death. This seems very strange and upsetting until you consider what these upsetting thoughts cause the person to do. They cause the person to move away from the edge so they won’t fall! From this viewpoint, damnation is your personal, spiritual cliff, and your brain plays a scary movie to help keep you from falling off.*

*“Now I’d like you to do your best to restate what I just said about the safety-brain and how it relates to your upsetting thoughts about damnation and Hell. Even if you’re unsure what you believe, do your best to make the same case I just made, but in your own words.”*

**Repeat Imaginal Exposure to Blasphemous Thoughts**

The therapist repeats the imaginal exposure.

**Homework**

The patient is instructed to listen to the entire audiotape of the exposure session (including new information) twice daily while recording subjective distress and degree of perceived sinfulness indicated by having blasphemous thoughts before and after each listening exercise. The patient is instructed to avoid saying the ritual prayer and to record any instances where this happens.

## 2 Putative non-extinction processes

The clinical vignette describes a common course of treatment in our OCD clinic, which includes: (1) repeated exposure to available elements of the feared outcome (e.g., being consigned to Hell), fear triggers (e.g., blasphemous statements) and response prevention (no forgiveness prayer), and (2) introduction of new information regarding the meaning of the patient’s obsessive fears that occurs during the exposure. Note that these intervention steps did nothing to disconfirm the patient’s core fear of being consigned to Hell after death. In this section, we discuss two psychological therapeutic change processes putatively engaged by these procedures, and their potential relevance for exposure therapy across a broad range of cases.

### 2.1 Non-associative habituation

Nothing about the intervention described in the vignette could have demonstrated that blasphemous thoughts do or do not result in damnation after death. As such, it is reasonable to assert that extinction learning concerning the association of blasphemous thoughts with damnation would not account for any clinical benefit obtained (Note that while the case described is generic, we have commonly treated successfully similar cases in our clinic). In the absence of extinction learning, a potential driver of fear reduction through exposure to the feared outcome (in imagination, in this case) is *non-associative habituation*^1^, also known as single-stimulus habituation, a process by which physiological and psychological reactions to an evocative stimulus attenuate with repeated presentations (Table 1). Whereas repeated presentation of a learned threat-cue in the absence of the expected aversive outcome leads to an *associative* decrement in fear via extinction learning, the repeated presentation of the aversive stimulus itself can lead to *non-associative* habituation of fear responding. Thus, when exposure exercises involve repeated presentations of an aversive stimulus (note, this could include either fear triggers or the feared outcome itself), a decrease in the patient’s reactivity to the aversive stimulus across repeated presentations would be expected to occur via non-associative habituation (see also: Kredlow et al., 2022).

The vignette illustrates how we have modified our practice of ExRP to promote fear reduction via non-associative habituation. Not only does the imaginal exposure include the presumed fear triggers (blasphemous thoughts), it also includes imaginal elements of the feared outcome (e.g., the actual experience of being in Hell after death). This is likely to elicit high levels of fear while also forcing the patient to confront the certainty or uncertainty of damnation after death; prior to omitting the forgiveness prayer, the certainty of damnation would have remained a hypothetical question only. In our practice, this potent combination of aversive experiences – an imagined experience of damnation, and the possibility of actual damnation in the future – is repeatedly presented to the patient with the expectation that both will become less aversive with repeated presentations due to non-associative habituation.

It is important to note that this practice (i.e., exposure and ritual prevention in the absence of disconfirmation of the feared outcome), while common in our clinical practice and likely others’, is inconsistent with a purely extinction-based model, given that extinction learning requires presentation of the presumed CS+ while explicitly withholding the presumed US (see Figure 1, column A). The principle that exposure to feared outcomes and triggers – *in-vivo* or in imagination – can reduce the intensity of fear responding, even in the absence of disconfirmation, crucially informs exposure planning in many cases. The imaginal exposure exercise is designed to closely approximate the feared outcome itself, and constitutes a highly aversive experience of images, thoughts, and emotions associated with this outcome.

In this conceptualization of exposure, there is still room for extinction learning where expectations are violated. For example, if the patient over-estimates the level of distress the imaginal exposure would produce, therapeutic benefit via extinction learning would be expected (Jacoby and Abramowitz, 2016). In many cases, however, the patient instead makes correct predictions about the initial distress level of the imaginal exposure. Over multiple exposures to the feared outcome (in imagination in this case), non-associative habituation should lead the patient to experience decreasing aversiveness, which could *then* serve as an expectancy violation promoting extinction learning. For example, the patient might initially predict that the imaginal exposure will be highly uncomfortable, or that the uncertainty will be very difficult to tolerate, and then find this to be true in the first several exposure exercises. After repeated exposures, the patient might find that the images, and/or the accompanying uncertainty, are newly tolerable. This could pave the way for new extinction learning about the tolerability of feared thoughts or images (e.g., Jacoby and Abramowitz, 2016) or about the tolerability of uncertainty (e.g., Dugas et al., 2022). Importantly, however, non-associative habituation would still serve as the necessary first ingredient in obtaining this result.

### 2.2 Leveraging exposures to incorporate new therapeutic information

Extinction learning provides *corrective information*, acquired through experience, which therapeutically modifies a fear association; i.e., that the feared outcome does not follow the presentation of the fear trigger, even in the absence of rituals. We posit here that corrective information may also be introduced through non-experiential semantic or informational learning in the context of exposure. The vignette illustrates an example of this: the therapist provides information aimed at generating a competing non-fear inducing interpretation of the meaning of the patient’s intrusive thoughts; i.e., that the intrusive thoughts of being consigned to Hell represent the brain’s effort to deter the patient from sin rather than constituting a sin itself. This viewpoint is in line with several established theoretical accounts that acknowledge a role for both experiential and semantic learning in the acquisition of threat and safety contingencies: e.g., Lovibond (2006) propositional expectancy model (an extension of Seligman and Johnston (1973) cognitive learning model), in which learning represents an accumulation of propositional knowledge that may be acquired via multiple means. According to this model, in the vignette, modification of the patient’s obsessional belief could be achieved through a combination of semantic learning (e.g., conveying through language that blasphemous thoughts in the absence of a ritual might not guarantee damnation), and experiential learning (e.g., the patient experiencing that they can tolerate the distress associated with exposures). Moreover, findings show that new information is more memorable when presented in an emotionally evocative context (Christianson, 1992; Holland and Kensinger, 2010) and that affective arousal during therapy sessions is a key predictor of psychotherapy efficacy (Frank and Frank, 1993; David and Szentagotai, 2006; Markowitz and Milrod, 2011), suggesting that exposure exercises may offer a particularly fruitful opportunity for conveying therapeutic information.

The practice of leveraging emotional arousal during exposures to enable therapeutic learning can also be aligned with Foa and Kozak’s (1986) emotional processing theory, as well as recent findings and theory related to memory reconsolidation. Regarding the former, Foa and Kozak (1986) cite evidence that fear excitation during and just after exposures is a critical pre-requisite to successful exposure, and they theorize that this is required for the incorporation of corrective information into the patient’s pathological “fear structure.” (The fear structure is conceptualized as an interconnecting network of stored information about feared stimuli, outcomes, responses, and their meaning.) In line with this, memory reconsolidation theory [review: (Lee et al., 2017); though see also: (Schroyens et al., 2023)] holds that established memories are uniquely amenable to modification during their reactivation (or retrieval) into working memory. Specifically, reactivation of a stored memory is thought to induce a brief labile period, lasting seconds to hours, during which new information may be incorporated with the original memory upon reconsolidation of the activated memory (Lewis, 1979; Nader, 2003).

In our clinical vignette, the therapist verbally conveys new therapeutic information about the meaning of the patient’s obsessional content, including: (a) the perspective that intrusive thoughts stem from the brain’s “primitive warning system” to prevent rather than promote harmful outcomes (damnation, in this case); and, (b) the consistency of this therapeutic meaning with past experience (ego dystonic images of jumping off a bridge to induce moving away from the edge for safety). The therapist provides this new therapeutic information at a targeted moment: just after the patient completes the first iteration of the imaginal exposure and is experiencing strong emotional arousal, and just prior to a repetition of the imaginal exposure. This timing is meant to ensure that the new information is introduced during the labile period brought about via reactivation of the fear structure. Thus, the therapist induces and then capitalizes on a period of fear-related memory instability as a means of introducing new information into the fear memory network prior to reconsolidation into long-term memory.

The practice of introducing corrective information in the context of exposure must include several considerations. Introducing such information too early in the exposure exercise may prevent the patient from reaching and maintaining sufficient emotional arousal for the established fear structure to be adequately activated and labile, thus inhibiting the integration of the corrective information via reconsolidation into long-term memory. On the other hand, simply activating the fear memory while conveying little or no new corrective information (e.g., devoid of both disconfirmation and other therapeutic information) risks leaving the pathological fear structure largely intact upon its reconsolidation. This could pose an ethically problematic scenario in which the patient would be made upset through the exposure while achieving no fundamental therapeutic change.

It is important to note distinctions between the approach described here and conventional cognitive restructuring, a commonly used technique for addressing distorted or inaccurate beliefs. To date, cognitive restructuring techniques, i.e., identifying and challenging faulty appraisals, have been emphasized as a treatment modality distinct from exposure (Kaczkurkin and Foa, 2022) or as a component of post-exposure processing (McLean and Foa, 2013). Clinical trials have indicated that traditional cognitive restructuring can be moderately helpful in the treatment of OCD (Freeston et al., 1997; McLean et al., 2001), but add no advantage in terms of treatment efficiency (Vogel et al., 2004). Importantly, the technique illustrated in our vignette differs in key ways from traditional cognitive restructuring, as well as from post-exposure processing. First, we position the introduction of therapeutic information within the ongoing exposure to promote its incorporation into the fear memory structure upon reconsolidation. This can be contrasted with traditional cognitive restructuring and post-exposure processing, which occur outside of exposure. Our approach is further distinguished from traditional cognitive restructuring and post-exposure processing in the extent to which the therapist is responsible for the content of the new information provided. We view the generation of therapeutic content in this context to be uniquely within the purview of the trained therapist’s expertise, unlike traditional Socratic questioning methods which eschew therapist-generated interpretations. In our vignette, for example, the patient is not expected to generate the alternative meaning of symptoms provided by the therapist.

It has also been documented that efforts at inducing cognitive change during an exposure that provide *reassurance* to the patient can interfere with therapeutic progress by blocking direct exposure to the feared situation and its accompanying uncertainty (Gillihan et al., 2012). In our vignette, the therapist is careful to inject the new information only at the key moment when fears are fully activated during exposure. The therapist then repeats the exposure after having introduced the new therapeutic information. Crucially, the new therapeutic information is structured to avoid frank reassurance; i.e., it does not include statements such as “You will not go to Hell,” or “There is no Hell.” Rather, the therapist aims to provide a plausible alternative meaning for the patient’s blasphemous thoughts. It would also be important for the therapist to not respond to requests for reassurance the patient might make (e.g., “Are you sure that having these thoughts aren’t a sin?”). These steps are designed to prevent the new information from serving as reassurance that blocks therapeutic gains.

## 3 Discussion

**Figure 2:**
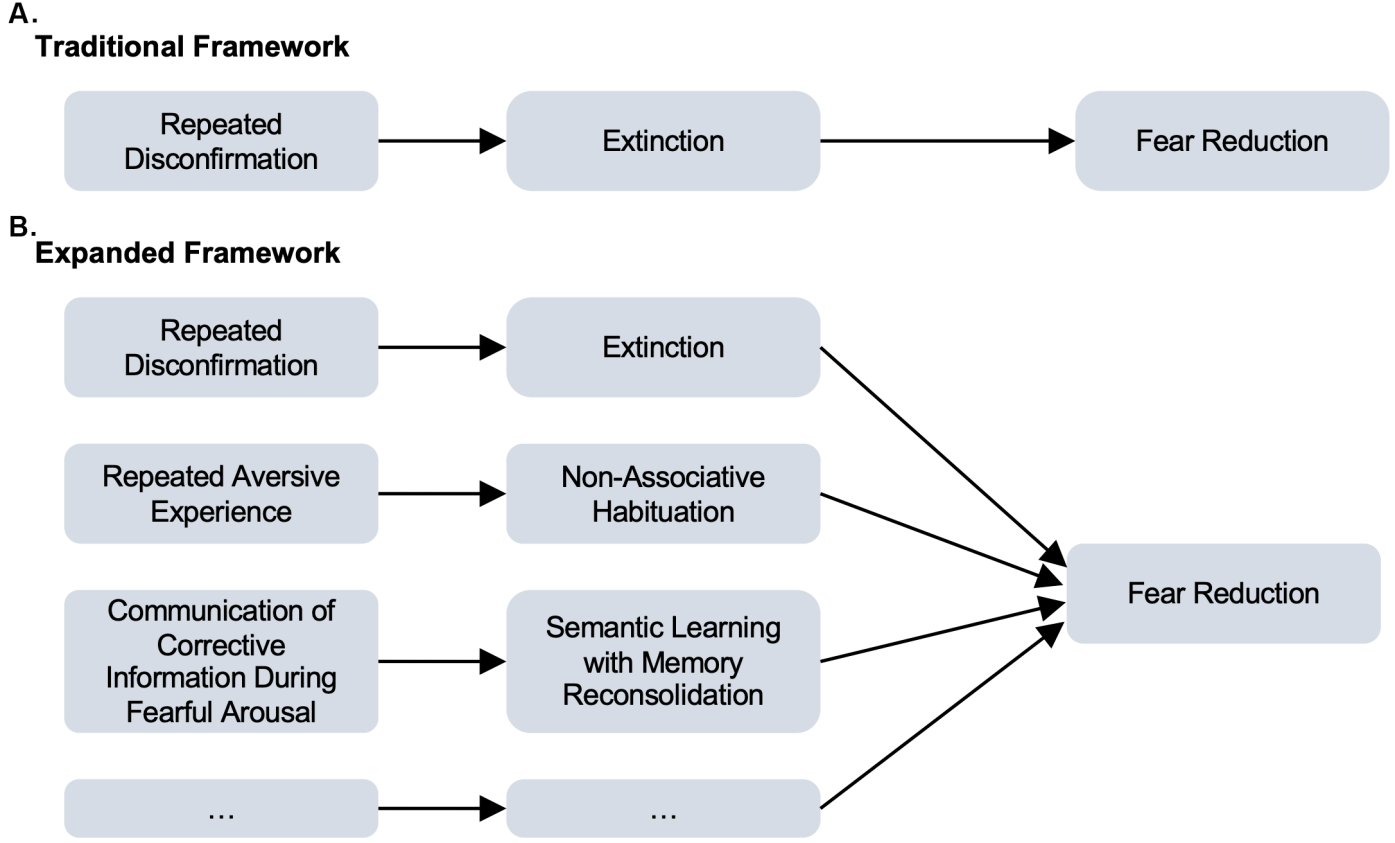
The traditional framework centers on extinction learning as a means to fear reduction. Our expanded framework includes extinction learning as one of many mechanisms by which exposure therapy can bring about fear reduction.

In our practice of treating OCD through exposure therapy, we have developed and successfully used strategies designed to leverage psychological processes in addition to extinction learning, especially (but not exclusively) for patients whose primary fears are not amenable to disconfirmation. As described here, we assert that the success of these strategies is not accounted for by traditional extinction-focused formulations, which requires disconfirmation of the feared outcome. Based on this and our success in treating patients whose feared outcomes cannot be disconfirmed, we argue that an exclusive focus on extinction learning in conceptualizing and executing exposure interventions fails to harness the full potential of this therapeutic tool. Figure 2 summarizes our expanded view of exposure therapy’s efficacy, listing the various processes, in addition to extinction learning, that may lead to fear reduction resulting from exposure.

In this paper, we describe two non-extinction based practices that we have routinely and successfully employed in our clinic. The first of these entails the repeated exposure to fear triggers and feared outcomes (whether *in-vivo* or in imagination) in the absence of disconfirmation of the feared outcome and rituals. Non-associative habituation (i.e., decrements in the intensity of stimulus responses with repeated presentations) provides a known mechanism that can explain the efficacy of this therapeutic practice. The second is the practice of strategically conveying therapeutic information to the patient during exposure with the goal of incorporating this new therapeutic information into the exposure-activated fear structure upon its reconsolidation into long-term memory storage.

While we have focused in this paper on a common presentation of OCD that underscores the limitations of the extinction model and illustrates our non-extinction strategies, we believe these strategies could be beneficial in treating a wide range of patient presentations, even when some degree of disconfirmation through exposure is feasible (e.g., see Figure 1, column C), in OCD and other conditions. For example, a patient with social anxiety disorder who fears public speaking might experience disconfirmation of some feared outcomes through exposure (e.g., “I will pass out”), but not others (e.g., “the audience believes I am terrible at my job”). We posit that such a case would benefit from the introduction of new therapeutic information in the context of fear activation through exposure (e.g., “your brain imagines these feared scenarios not because they are highly likely, but because you care deeply about preventing them”). As the patient advances from imaginal exposure to *in vivo* exposures, the patient and therapist might collaborate on ways for the patient to retrieve the new information at key moments during the exposure, deepening the new memory structure. These strategies could be applied alongside traditional exposure techniques that target extinction learning.

Existing experimental paradigms that elicit non-associative habituation (e.g., Harris, 1943) and memory modification in the process of reconsolidation (e.g., Nader et al., 2000) can be used to test these strategies. Experimental research, for example, could investigate lab-based analogues for these exposure strategies as a means of informing the continued development and refinement of clinical techniques. Research with human participants rather than non-human animals is of particular value in this area, especially in terms of studying the effects of semantic learning in the context of exposure. For example, clinical trials could investigate whether strategic delivery of therapeutic information during an exposure strengthens (as we suggest it should) or weakens (as inhibitory learning theories presumably suggest) the effectiveness of exposure.

### 3.1 Limitations

As a reverse translational endeavor, this work seeks to inspire new ideas and research stemming from our clinical experience and practices. As such, we cannot make strong empirical claims as to the validity of our conjectures. The “bedside-to-bench” trajectory of discovery has a history of advancing medical science (Moore, 2008; Shakhnovich, 2018; Wagner, 2018), including psychiatry (Malkesman et al., 2009; Daniels et al., 2020), but this depends on subsequent empirical investigation of the treatment targets and therapeutic techniques proposed. The clinical utility of the ideas presented here thus remains to be empirically tested. Another limitation of this work is its narrow focus on two specific clinical processes underlying non-extinction-based effects of exposure therapy. We believe it is likely that that other non-extinction processes warrant elaboration and closer inquiry. For example, exposure in the absence of disconfirmation of the feared outcome could induce cognitive dissonance that is resolved in part by reassessing either the likelihood or severity of the feared outcome. Exposure with response prevention could also induce learned helplessness concerning the feared outcome; i.e., the acceptance of the lack of control over feared outcome. These and other non-extinction processes warrant empirical investigation regarding their potential role in clinical fear reduction.

### 3.2 Conclusion

A successful course of exposure treatment can yield life-changing symptom relief, whereas an unsuccessful course of treatment can leave patients frustrated, confused, and even discouraged from pursuing future mental health care. We describe specific techniques and putative mechanisms of change which may be related to positive therapeutic outcomes in exposure therapy with patients for whom extinction learning is definitionally limited or precluded; i.e., those whose feared outcomes cannot be disconfirmed in the context of exposure. A continued effort to advance the framework for exposure therapy’s mechanisms of action, through experimental research and clinical development, holds promise to improve clinical outcomes. We hope that the clinical observations and ideas outlined in this paper can be used to inform the field, improve the efficacy of clinical practice and spur new empirical research.

## Data availability statement

The original contributions presented in the study are included in the article/Supplementary material, further inquiries can be directed to the corresponding author.

## Author contributions

HB: Conceptualization, Visualization, Writing – original draft, Writing – review & editing. RW: Conceptualization, Visualization, Writing – review & editing. SK: Conceptualization, Writing – review & editing. MK: Conceptualization, Writing – review & editing.

## Footnotes

^1^Of note, the term “habituation” has been used elsewhere to refer broadly to fear decrements during exposures (e.g., Seligman and Johnston, 1973; Jacoby and Abramowitz, 2016), both within and between exposures, which can include both associative and non-associative elements. Here, however, we refer specifically to non-associative habituation; that is, decrements in aversive stimulus responses that are not attributable to any change in the stimulus’s association with other stimuli.
